# Non-Invasive Respiratory Impedance Enhances Cerebral Perfusion in Healthy Adults

**DOI:** 10.3389/fneur.2017.00045

**Published:** 2017-02-16

**Authors:** Christopher G. Favilla, Ashwin B. Parthasarathy, John A. Detre, Arjun G. Yodh, Michael T. Mullen, Scott E. Kasner, Kimberly Gannon, Steven R. Messé

**Affiliations:** ^1^Department of Neurology, University of Pennsylvania, Philadelphia, PA, USA; ^2^Department of Physics and Astronomy, University of Pennsylvania, Philadelphia, PA, USA; ^3^Department of Radiology, University of Pennsylvania, Philadelphia, PA, USA

**Keywords:** cerebral blood flow, cerebral blood flow measurement, cerebral hemodynamics, near-infrared spectroscopy, transcranial Doppler, diffuse correlation spectroscopy, respiratory impedance

## Abstract

Optimization of cerebral blood flow (CBF) is the cornerstone of clinical management in a number of neurologic diseases, most notably ischemic stroke. Intrathoracic pressure influences cardiac output and has the potential to impact CBF. Here, we aim to quantify cerebral hemodynamic changes in response to increased respiratory impedance (RI) using a non-invasive respiratory device. We measured cerebral perfusion under varying levels of RI (6 cm H_2_O, 9 cm H_2_O, and 12 cm H_2_O) in 20 healthy volunteers. Simultaneous measurements of microvascular CBF and middle cerebral artery mean flow velocity (MFV), respectively, were performed with optical diffuse correlation spectroscopy and transcranial Doppler ultrasound. At a high level of RI, MFV increased by 6.4% compared to baseline (*p* = 0.004), but changes in cortical CBF were non-significant. In a multivariable linear regression model accounting for end-tidal CO_2_, RI was associated with increases in both MFV (coefficient: 0.49, *p* < 0.001) and cortical CBF (coefficient: 0.13, *p* < 0.001), although the magnitude of the effect was small. Manipulating intrathoracic pressure *via* non-invasive RI was well tolerated and produced a small but measurable increase in cerebral perfusion in healthy individuals. Future studies in acute ischemic stroke patients with impaired cerebral autoregulation are warranted in order to assess whether RI is feasible as a novel non-invasive therapy for stroke.

## Introduction

Optimization of cerebral blood flow (CBF) is a cornerstone of clinical management for a number of neurological diseases that result in impaired oxygen delivery to the brain, most notably ischemic stroke. A decrease in intrathoracic pressure has been shown to increase cerebral perfusion in animal models of cardiac arrest and hypotension, but human data are limited (1–4). These effects are likely driven by an increase in venous return to the heart (5, 6), which in turn promotes cardiac output (CO) (6, 7) and blood pressure (1, 7). While intrathoracic pressure can be manipulated during mechanical ventilation, a non-invasive approach can have a similar effect in spontaneously breathing patients. A respiratory device incorporating a one-way valve that provides resistance only during the inspiratory component of the respiratory cycle, referred to as respiratory impedance (RI), leads to augmentation of the inspiratory effort in order to generate enough negative intrathoracic pressure to overcome the impedance. This device has typically been used for respiratory muscle training, but the effect on cerebral perfusion in humans is not well studied. The limited data that exist have focused on patients with orthostatic hypotension, where RI has been shown to increase CBF velocity [measured by transcranial Doppler (TCD)] (1), and reduce subjective symptoms (1, 8).

Transcranial Doppler provides an important measure of cerebral hemodynamics, capturing blood flow velocity through proximal intracranial vessels. This non-invasive, continuous measure provides a valuable surrogate to global CBF. Diffuse correlation spectroscopy (DCS) is a relatively new optical technique that permits real-time, continuous, non-invasive bedside monitoring of tissue-level CBF using near-infrared light (9–12). DCS holds great promise for monitoring cerebral hemodynamics (13, 14) and has been validated against other measures of CBF such as ASL-MRI (15), Xenon CT (16), TCD (17), phase-encoded velocity mapping MRI (18), and fluorescent microspheres (19). This instrumentation has also been recently employed to quantify changes in CBF associated with position change after stroke (20). For this study, we use both DCS and TCD to measure changes in cerebral hemodynamics that occur during RI in healthy adults.

## Materials and Methods

### Study Population

Twenty healthy adult volunteers were enrolled in this study at the Hospital of the University of Pennsylvania between August 2015 and October 2015. Subjects were eligible for the study if they were over 18 years of age but were excluded if any of the following were present: history of stroke or transient ischemic attack, known cerebrovascular disease, history of congestive heart failure, history of COPD, prior neurosurgical procedure, history of brain tumor, or active pregnancy. Subjects with well-controlled vascular risk factors, such as hypertension and hyperlipidemia, were permitted to participate in the study. The protocol was approved by the University of Pennsylvania Institutional Review Board (Protocol Number 822204). Written informed consent was signed by each participant prior to enrollment.

### CBF Monitoring

**Figure 1:**
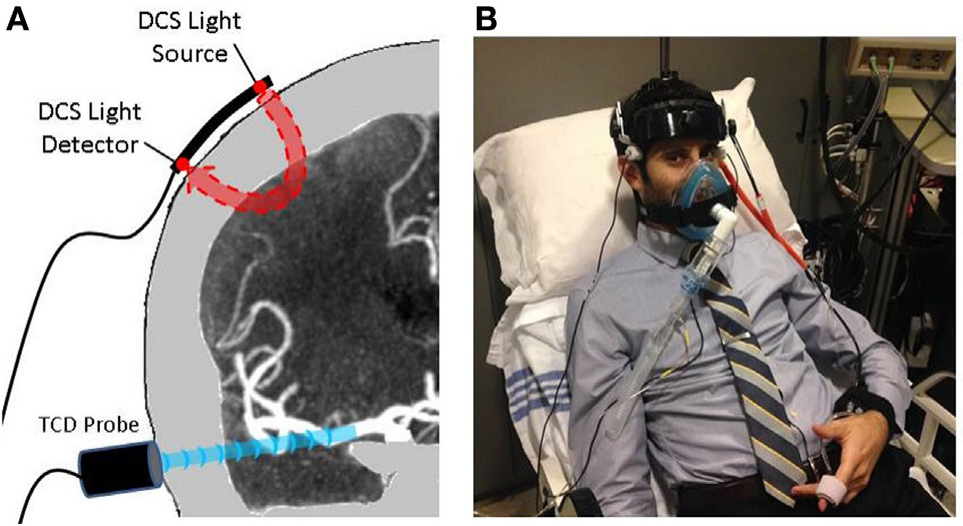
**Protocol setup: (A) transcutaneous cortical cerebral blood flow monitoring by diffuse correlation spectroscopy (DCS) and middle cerebral artery trunk velocity monitoring by transcranial Doppler (TCD)**. **(B)** A subject prepared for the protocol with all cerebrovascular and cardiopulmonary equipment in place.

Diffuse correlation spectroscopy provides a transcranial measurement of relative CBF. Briefly, the temporal fluctuations of near-infrared light scattered by moving red blood cells in tissue are detected. These fluctuations are quantified by the light intensity temporal autocorrelation function. Its decay rate is related to changes in CBF (21). Our instrument employed a long-coherence-length laser operating at 785 nm and four single-photon counting avalanche photodiode detectors for each hemisphere (i.e., a total of two lasers and eight detectors). Optical fibers were used to couple sources and detectors to the head *via* 2 cm × 5 cm rubber optical probes that were placed bilaterally at the temporal margin of the forehead, superior to the frontal sinuses; this configuration enabled measurement of tissues supplied by the anterior middle cerebral artery (MCA). The distance between the source and detector fibers was 2.5 cm, permitting average light penetration through the cortical surface (~1.25 cm). An elastic headband was placed over the optical probes to maintain secure contact during the course of the protocol (Figure 1). Data were collected from both hemispheres at a sampling frequency of 1 Hz. Mean CBF was calculated for each segment of the protocol after discarding 10 s preceding and 20 s following each RI transition. This protocol helped to negate spurious motion-induced signal fluctuations and enabled stabilization after each transition.

### Blood Flow Velocity Monitoring

Mean flow velocity (MFV) within the MCA was assessed in all subjects using a Compumetics DWL TCD System (Compumetics Ltd., Singen, Germany). Probes were secured using a DiaMon^®^ adjustable headframe (Figure 1). MCA trunks were insonated bilaterally *via* transtemporal windows at a depth of 40–65 mm. Emphasis was placed on obtaining reliable signal from one MCA, as there was no expectation of asymmetry in this population of healthy volunteers. MFV waveforms were sampled at a rate of 25 Hz, time-synchronized, and recorded on a computer with DCS-measured CBF. Average values were calculated for each segment of the protocol, i.e., after discarding 10 s preceding and 20 s following each RI transition. This protocol helped to negate spurious motion-induced signal fluctuations and enabled stabilization after each transition.

### Cardiopulmonary Monitoring

A finger photophlethysmogaph (Finapres Medical Systems, Arnhem, Netherlands) was placed on the right wrist and third digit of the right hand and provided continuous measurement of mean arterial pressure (MAP), systolic blood pressure, heart rate (HR), and CO. A transcutaneous pulse-oximeter was placed on the second digit of the right hand for continuous measurement of oxygen saturation. The RI device was coupled with a sensor that provided continuous measurements of both end-tidal CO_2_ and respiratory rate (Figure 1). All cardiopulmonary waveforms were digitized, time-synchronized, and recorded on a computer with DCS-measured CBF at a sampling frequency of 25 Hz. Average values were calculated for each segment of the protocol after discarding 10 s preceding and 20 s following each RI transition, in order to negate spurious motion-induced signal fluctuations and enable stabilization after each transition.

### RI Protocol

The Philips Inspiratory Muscle Trainer (IMT; Philips Respironics) was utilized to non-invasively augment RI. The device has a one-way, spring-loaded valve, which provides an adjustable resistance during inspiration only. No resistance is imposed during expiration. When the device is in place, inspiratory effort must increase in order to generate sufficient negative intrathoracic pressure to overcome the selected resistance. Three discrete levels of resistance were tested (6 cm H_2_O, 9 cm H_2_O, and 12 cm H_2_O). Every subject was exposed to all three levels of resistance in a prespecified random order.

Each subject was positioned in a hospital bed, with the head-of-bed at 45°. Baseline hemodynamic data were collected for 5 min, during which the subject was breathing through a respiratory mouthpiece providing no resistance. The IMT was then mounted to the back of the respiratory mouthpiece for a 3-min RI segment, during which the subject was instructed to breathe naturally through the IMT and maintain a relatively stable respiratory rate, in order to avoid fluctuations in end-tidal CO_2_, if possible. If fluctuation in respiratory rate or end-tidal CO_2_ occurred, the subject was reminded to breathe comfortably at a normal rate, but more intensive attempts to coach breathing were avoided. After 3 min of RI, the IMT was removed for 3 min. This 6-min cycle was repeated for each level of resistance. Subjects were blinded to the level of resistance. At the completion of the study protocol, subjects were asked if they experienced shortness of breath, chest pain, fatigue, and lightheadedness. A single Neurologist at the Hospital of the University of Pennsylvania was familiar with the protocol and present for the entirety of the protocol. The protocol was carried out in the CBF lab, within the Hospital of the University of Pennsylvania.

### Statistical Analyses

All data processing was performed while blinded to the level of RI. Mean cerebral perfusion (using both CBF and MFV) values for each RI segment were compared to the preceding 3 min of normal breathing. Pairwise comparisons were completed using the Wilcoxon signed-rank tests. Kruskal–Wallis test and Cuzick’s non-parametric test of trend were used to compare perfusion measures across all RI segments. Additionally, mixed-effects linear regression was employed, using a maximum likelihood to model changes in DCS and TCD across levels of RI. Models incorporated a random slope, and the covariance was modeled as unstructured. This approach was used in a prior study of DCS–CBF and head-of-bed manipulation (20). DCS and TCD were dependent variables. Level of RI was the independent variable and was considered to be an interval variable. The subject variable was included in the model to assess possible individual variability. End-tidal CO_2_ was included in the model in order to account for possible device-related effects, which may influence perfusion, independent of the proposed mechanism. Specifically, subjects were encouraged to breathe at a normal rate, yet if the device caused them to hyperventilate, MFV and CBF would be expected to decrease. Blood pressure and CO were not included in the model because they are expected to be on the causal pathway, rather than confounders. In a secondary analysis, the mixed-effects regression was repeated without the inclusion of end-tidal CO_2_, and the level of RI was considered to be categorical rather than interval.

The sample size was derived from prior TCD data in human subjects (1), from which we estimated a 10% mean increase in MFV associated with RI (SD 10%). Setting power to 0.80 and significance to 0.05, 16 healthy controls would be sufficient to demonstrate the effect of the intervention. No previous literature exists to provide expectations of mean increase in DCS-derived CBF associated with RI. However, considering the relative similarity between perfusion changes measured with TCD and DCS (17, 20), we posit that the sample size calculations used for TCD measures will be applicable for CBF measured with DCS.

## Results

The study enrolled 20 consecutive healthy volunteers. The average age was 39 years (SD: 11 years). Fifty-five percent of volunteers were male, and 70% were Caucasian. Vascular risk factors were uncommon in the cohort: 10% had hypertension, 15% hyperlipidemia, 10% asthma, and no subjects had diabetes or coronary artery disease. No subjects were taking beta-blockers. Also, 5% were taking nodal acting calcium channel blockers, and 10% were taking inhaled bronchodilators. There were no adverse events associated with the IMT device. Specifically, no subjects reported shortness of breath, chest pain, fatigue, or lightheadedness. No subjects elected to terminate the protocol before completion, and there were no documented episodes of hypoxia, hypoventilation, or hyperventilation.

**Figure 2:**
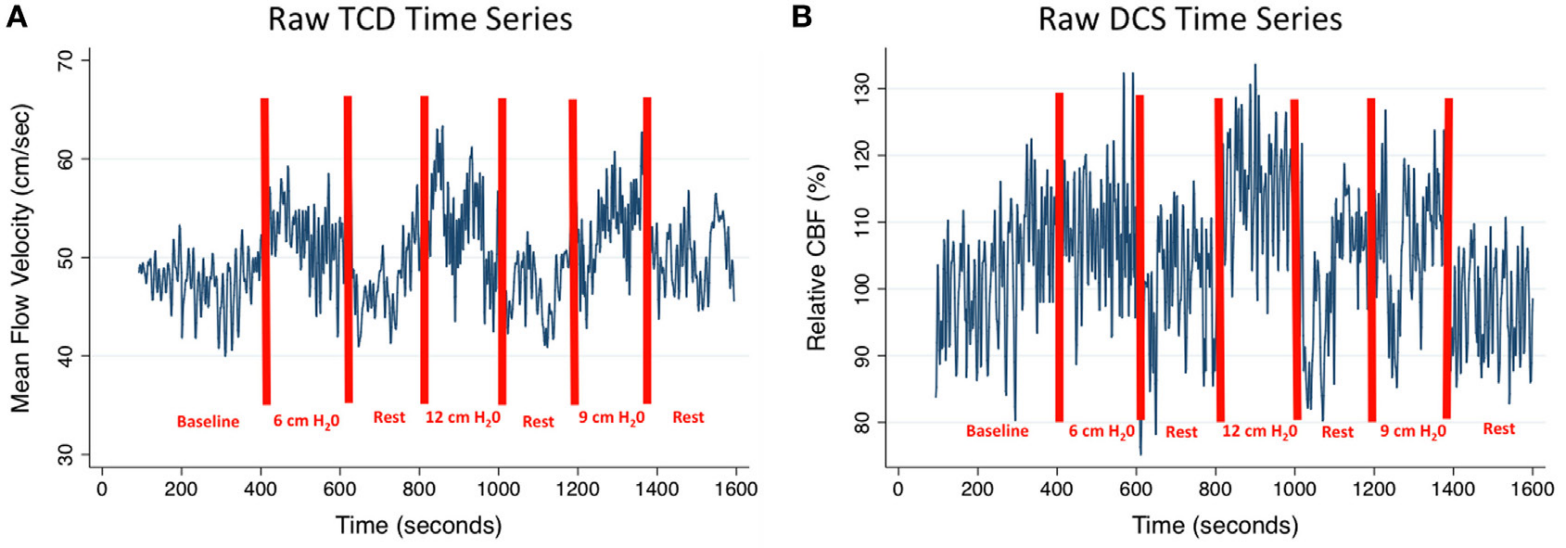
**CBF and MFV time series: an example of hemodynamic changes throughout the protocol for one subject, (A) middle cerebral artery MFV and (B) cortical CBF**. CBF, cerebral blood flow; TCD, transcranial Doppler; DCS, diffuse correlation spectroscopy; MFV, mean flow velocity.

**Figure 3:**
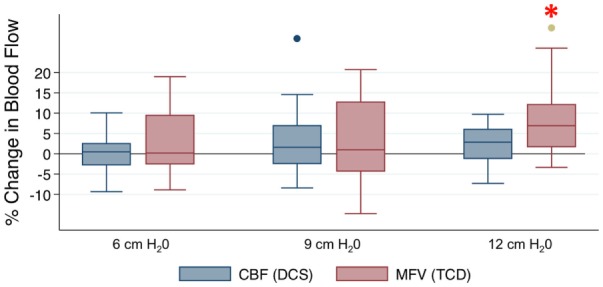
**The effect of respiratory impedance on cerebral perfusion: box-and-whisker plots represent medians, inter-quartile ranges, and the full range of the data**. The two additionally plotted points represent outliers. *p < 0.005 by Wilcoxon signed-rank tests. CBF, cerebral blood flow; DCS, diffuse correlation spectroscopy; TCD, transcranial Doppler; MFV, mean flow velocity.

Figure 2 provides an example of the raw time series data acquired from the study of one subject, where there is augmentation of both cortical CBF and MCA flow velocity during each level of RI, most notable with the highest level of RI. Figure 3 depicts the relationship between blood flow and RI averaged across the cohort. There was a 6.4% increase in TCD-measured MFV at the maximum level of resistance (12 cm H_2_O) (*p* = 0.004), but no significant change from baseline was noted at the low and medium levels of RI. Pairwise testing similarly compared DCS-measured CBF at each level of RI (in comparison to baseline), and while no significant differences were identified, point estimates suggest a subtle dose–response relationship between CBF and RI. Table 1 depicts all hemodynamic data, including MAP, HR, CO, and end-tidal CO_2_ across the range of RI. Similar to the change in MFV, an increase in MAP was noted with the highest level of RI. Importantly, when averaged over the cohort, there were no significant changes in end-tidal CO_2_.

**Table 1:** **Hemodynamic changes with respiratory impedance (RI): % changes relative to baseline**.

	6 cm H_2_O	*p*-Value	9 cm H_2_O	*p*-Value	12 cm H_2_O	*p*-Value	*p* for trend
Cerebral blood flow (diffuse correlation spectroscopy)	+0.5% (−2.7 to 2.5)	0.82	+1.6% (−2.4 to +6.9)	0.18	+2.7% (−3.4 to +5.4)	0.46	<0.001
Blood flow velocity (transcranial Doppler)	+0.2% (−2.5 to +9.4)	0.23	−0.1% (−4.7 to 11.7)	0.79	+6.4% (+1.1 to +11.0)	0.004	<0.001
Mean arterial pressure	+1.0% (−1.9 to +2.9)	0.22	+0.3% (−0.6 to +2.5)	0.25	+1.7% (+0.6 to +3.5)	0.006	0.005
Heart rate	+1.7% (−2.9 to +4.3)	0.33	+1.8% (−1.9 to +5.8)	0.13	+0.3% (−3.6 to +2.9)	0.88	0.36
End-tidal CO_2_	−2.8% (−8.5 to +0.1)	0.10	−1.0% (−10.0 to +3.5)	0.30	+1.2% (−1.6 to +7.3)	0.23	0.90
Cardiac output	−0.1% (−1.7 to +1.0)	0.77	+1.1% (−0.9 to +2.5)	0.22	+0.8% (−1.1 to +2.4)	0.25	0.17

*All reported values are median and IQR. All values are reported as percentages, relative to the baseline period immediately preceding RI. *p*-Values were calculated by Wilcoxon signed-rank tests, in comparison to baseline values (3-min rest period immediately prior to each intervention). Non-parametric test for trend was performed across ordered groups and reported in the final column*.

When all levels of RI were compared by Kruskal–Wallis test, both TCD and DCS were significantly different across levels (*p* < 0.001 for both measures). A Cuzick’s non-parametric test of trend confirmed that this difference across levels of RI was ordered for both TCD and DCS (*p* < 0.001 for both measures). To better quantify the trend, mixed-effects linear regression was employed, incorporating end-tidal CO_2_ and individual variability in the model, this approach demonstrated that level of RI was associated with both TCD-measured MFV (coefficient: 0.49, *p* < 0.001) and DCS-measured microvascular CBF (coefficient: 0.13, *p* < 0.001). The larger coefficient for MFV suggests that RI had a greater effect on flow through the intracranial trunk vessels as compared to cortical tissue perfusion, although the magnitude of effect was small overall. Post-estimation predicted probabilities suggest that RI of 12 cm H_2_O, relative to no RI, was associated with a 5.8% increase in TCD MFV and a 1.2% increase in CBF. Values predicted by the linear model were very similar to measured values at 6 cm H_2_O, 9 cm H_2_O, and 12 cm H_2_O. Removal of end-tidal CO_2_ did not influence the model for TCD or DCS. In the mixed-effects models, a Wald test identified significant variability between individuals for both TCD (*p* < 0.001) and DCS (*p* < 0.001). In a secondary analysis, the level of RI was considered to be categorical rather than interval, but the model was not significantly affected.

## Discussion

Non-invasive RI proved to be well tolerated and holds promise as a bedside intervention to augment cerebral perfusion. In this study of healthy volunteers, RI resulted in a small but significant increase in MFV measured by TCD. Brain tissue flow measured by DCS did not show a significant change as compared to baseline, but there appears to be a linear relationship between the level of RI and the resultant increase in both MCA trunk and tissue-level flow, independent of end-tidal CO_2_, although the magnitude of change was small. Changes in end-tidal CO_2_ are proportional to changes in CBF through its potent effect of vasomotor control (22, 23). Because RI influences the respiratory cycle, care was taken to quantify respiratory rate and end-tidal CO_2_ to ensure that the measured hemodynamic effects were a result of RI rather than a surrogate effect. While the effect of RI on TCD has been previously studied, the current study is the first to utilize measures of tissue-level cortical CBF and compare the perfusion response across three levels of RI.

In the comparison of individual medians, a significant increase from baseline flow was only noted by TCD at the highest level of resistance. This may indicate a threshold of effect, but more likely it is due to limited power to detect small differences. Similarly, DCS was unable to identify a significant change in tissue flow. The point estimates indicate a dose–response relationship, although, again, the absolute differences were small, and the study was not powered to detect these differences. The mixed-effects regression model for both TCD and DCS has greater power because it incorporates repeated measures for each subject. This model demonstrated a statistically significant increase in both DCS and TCD as RI increased, although the absolute difference was low.

The discrepancy between TCD and DCS may reflect the fact that the two technologies are monitoring different components of the vascular system. For example, the increase in MAP at the highest level of RI mirrored changes in MCA velocity. Cerebrovascular autoregulation, which is largely imposed at the arteriolar level (24, 25), may dampen the effect at the tissue level, resulting in a non-significant increase in DCS-measured CBF. Patient populations with impaired autoregulation may demonstrate greater effects of RI. While there are little human data available for comparison, a study of patients with orthostatic hypotension demonstrated a 10% increase in MFV with 7 cm H_2_O resistance (1), while a study of normovolemic healthy volunteers found that RI reduced symptoms generated by orthostatic maneuvers but yielded no objective effect on MFV (8).

Mechanistically, RI decreases intrathoracic pressure, which in turn increases venous return to the heart and ventricular preload (5, 6). Depending on volume status and cardiac function, the Frank–Starling law indicates that an increase in preload results in an increase in cardiac stroke volume, as previously observed (6, 7), which in turn increases vital organ perfusion, including brain perfusion (2, 26). This physiologic mechanism is more impactful in the context of hypovolemia (27, 28), and the modest effect measured in the current study may be a consequence of the subjects’ volume status. Additionally, as noted above, cerebral autoregulation may dampen the augmentation of tissue perfusion with RI. So, although we observed a small magnitude of effect in healthy volunteers, other populations may experience greater increases in CBF. For example, ischemic stroke impairs cerebrovascular autoregulation, and many patients with acute stroke present to the hospital in a hypovolemic state, which is associated with worse outcome (29, 30). As such, RI may achieve more significant increases in cerebral perfusion in patients with acute ischemic stroke, which will be an important area of future study.

Based on the proposed mechanism of increased cardiac preload, it may be surprising that RI did not increase CO, as was previously reported (31). It is worth noting that the Finapres continuous hemodynamic monitor does not directly measure cardiac stroke volume. Rather, it calculates stroke volume and CO based on the contour of the blood pressure waveform, so technical limitations should temper the confidence in this measurement. Still, prior studies of RI that have measured an increase in CO have used a similar approach (31, 32). The discrepancy between these findings may relate to the underlying volume status of the study subjects, but it would also be reasonable to consider more direct measure of stroke volume and CO in future studies.

An additional mechanism by which RI may increase cerebral perfusion is through manipulation of intracranial pressure (ICP). In animal models, decreases in ITP have been shown to result in reduced ICP (33, 34). This may occur because of the increased venous return through the jugular veins or spinal venous system. The potential relationship between ITP and ICP has not been studied in spontaneously breathing humans, and because the effect of interest in the current study was cerebral perfusion, an emphasis was placed on perfusion monitoring rather than a mechanistic evaluation, but potential mechanism could be explored in future studies.

Adjusting for end-tidal CO_2_ in the regression analysis did not diminish the effect of RI. Still, end-tidal CO_2_ should be a key component of future studies, because if a subject were to hyperventilate during RI, the resultant hypocapnia could be expected to influence vasomotor tone and flow, confounding results (22, 23). Over a longer period of time, which would be required for any kind of clinical utility, there is a theoretical possibility of respiratory fatigue, though this was not observed under the current protocol. RI was well tolerated, without any reported shortness of breath, lightheadedness, fatigue, or chest pain. Continuous pulse-oximetry demonstrated maintenance of appropriate blood oxygen saturation throughout the protocol. Low levels of RI have been shown to nearly double the work of normal physiologic breathing, without signs of intolerance, but again less than 10 min of RI was tested (35). Prior studies have exposed subjects to periods of RI greater than 10 min, with good tolerability (8, 31), showcasing the potential of using this intervention over longer time scales than tested in this study.

There are several limitations of the current study. Most notably, the detected changes in perfusion were smaller than anticipated, so the sample size was not powered to identify significant changes in perfusion during lower levels of RI. RI was limited to 3 min, so conclusions cannot directly be drawn about the effects of prolonged use. The volume status of each volunteer also serves as a significant confounder, and because volume status was not objectively quantified in this cohort, adjustment was not possible. Response to RI may be confounded by volume status, cardiac function, or dysrhythmia, which was not objectively assessed in this cohort. In addition, DCS signals cannot be reliably recorded through hair. Thus, tissue perfusion data are restricted to the frontal lobe, leaving other territories and deeper structures unmeasured. While TCD is a commonly used surrogate of CBF, it more specifically measures blood flow velocity through the MCA trunk, so one must assume a constant arterial diameter in order to use TCD as a reliable surrogate. This is a general limitation of TCD, but in the current study, we avoided such assumptions by also using DCS, a direct CBF monitor. A fairly homogenous population limited generalizability to specific patient populations, but the intent of this study was to demonstrate tolerability and potential for effect. Because the acute stroke population stands to benefit from an intervention that augments CBF, that population will be the subject of future study. In the study of stroke patients, attention should be paid to volume status, duration of RI, cardiac function, dysrhythmia, and infarct location.

## Conclusion

Manipulating intrathoracic pressure *via* non-invasive RI was well tolerated and resulted in a small but measurable increase in cerebral perfusion in healthy individuals. While flow changes were not identified at low levels of resistance, there was a linear relationship between the level of resistance and both MCA flow and tissue flow measures. Future study in ischemic stroke patients will assess whether RI has any utility as a novel non-invasive therapy for acute ischemic stroke treatment.

## Ethics Statement

This study was carried out in accordance with the Belmont Report with written informed consent from all subjects. All subjects gave written informed consent in accordance with the Declaration of Helsinki. The protocol was approved by the University of Pennsylvania Institutional Review Board (Protocol Number 822204).

## Author Contributions

CF designed the study, collected the data, interpreted the data, and drafted the manuscript. AP collected the data, analyzed the data, and revised the manuscript. JD designed the study, interpreted the study, and revised the manuscript. AY developed the technology, collected the data, and revised the manuscript. MM and SM analyzed the data, interpreted the data, and revised the manuscript. SK designed the study, interpreted the data, and revised the manuscript.

## Conflict of Interest Statement

Two authors, AY and JD, hold USA Patent #8082015, “Optical measurement of tissue blood flow hemodynamics and oxygenation” granted for the present class of DCS applications. Part of the technology has been transferred to a spin-off company (Hemophotonics, S.L., Barcelona, Spain), but the authors do not have any financial relationship to the company, do not have shares, and do not receive royalties. All other authors declare that they have no conflict of interest.
